# Application of Causal Inference to Genomic Analysis: Advances in Methodology

**DOI:** 10.3389/fgene.2018.00238

**Published:** 2018-07-10

**Authors:** Pengfei Hu, Rong Jiao, Li Jin, Momiao Xiong

**Affiliations:** ^1^Ministry of Education Key Laboratory of Contemporary Anthropology, School of Life Sciences, Fudan University, Shanghai, China; ^2^Department of Biostatistics and Data Science, School of Public Health, University of Texas Health Science Center at Houston, Houston, TX, United States; ^3^State Key Laboratory of Genetic Engineering, Collaborative Innovation Center for Genetics and Development, School of Life Sciences, Fudan University, Shanghai, China; ^4^Human Phenome Institute, Fudan University, Shanghai, China

**Keywords:** causal inference, genomic analysis, additive noise models for discrete variables, association analysis, entropy

## Abstract

The current paradigm of genomic studies of complex diseases is association and correlation analysis. Despite significant progress in dissecting the genetic architecture of complex diseases by genome-wide association studies (GWAS), the identified genetic variants by GWAS can only explain a small proportion of the heritability of complex diseases. A large fraction of genetic variants is still hidden. Association analysis has limited power to unravel mechanisms of complex diseases. It is time to shift the paradigm of genomic analysis from association analysis to causal inference. Causal inference is an essential component for the discovery of mechanism of diseases. This paper will review the major platforms of the genomic analysis in the past and discuss the perspectives of causal inference as a general framework of genomic analysis. In genomic data analysis, we usually consider four types of associations: association of discrete variables (DNA variation) with continuous variables (phenotypes and gene expressions), association of continuous variables (expressions, methylations, and imaging signals) with continuous variables (gene expressions, imaging signals, phenotypes, and physiological traits), association of discrete variables (DNA variation) with binary trait (disease status) and association of continuous variables (gene expressions, methylations, phenotypes, and imaging signals) with binary trait (disease status). In this paper, we will review algorithmic information theory as a general framework for causal discovery and the recent development of statistical methods for causal inference on discrete data, and discuss the possibility of extending the association analysis of discrete variable with disease to the causal analysis for discrete variable and disease.

## Introduction

By February 6th, 2017, a catalog of published genome-wide association studies (GWAS) had reported significant association of 26,791 SNPs with more than 1,704 traits in 2,337 publications (A catalog of Published Genome-Wide Association Studies, 2017)^1^. Many of these associated SNPs are non-coding variants (Timpson et al., 2017). Despite significant progress in dissecting the genetic architecture of complex diseases by GWAS, understanding the etiology and mechanism of complex diseases remains elusive. It is known that significant findings of association analysis are^1^ lacking in consistency and often proved to be controversial (Valente et al., 2015; Wakeford, 2015). Complex diseases are often caused by different genetic mutations, have complex and multimodal genetic etiology, and show substantial phenotype heterogeneity in morphology, physiology and behavior (Brookes and Robinson, 2015). There are multiple steps between genes and phenotypes. Each step may be influenced by genomic variation and can weaken links between genes and phenotypes. As a consequence, this will obscure the causal mechanisms of disease. The recent study finding “association signals tend to be spread across most of the genome” again shows that association signals provide limited information on causes of disease, which calls the future of the GWAS into question (Boyle et al., 2017; Callaway, 2017). Association and causation are different concepts (Altman and Krzywinski, 2015). Association of a genetic variant with the disease is to characterize the dependence between the genetic variant and disease, while causation from the genetic variant to the disease is to indicate that the presence of the genetic variant will produce effect and cause disease. Observed association may not infer a causal relationship and the lack of association may not be necessary to imply the absence of a causal relationship (Wakeford, 2015). Finding causal SNPs only by searching the set of associated SNPs may miss many causal variants and may not be an effective research direction in genetics. The dominant use of association analysis for genetic studies of complex diseases is a key issue that hampers the theoretical development of genomic science and its applications to discovery of mechanisms of diseases in practice. Causality shapes how we view and understand mechanism of complex diseases (Gottlieb, 2017). In addition, causal models can also be used to directly predict the results of intervention, but association usually cannot.

It is time to develop a new generation of genetic analysis to shift the current paradigm of genetic analysis from association analysis to causal inference. To make the shift feasible, we need (1) to develop novel causal inference methods for genome-wide and epigenome-wide causal studies of complex disease; (2) to develop unified frameworks for systematic casual analysis of integrated genomic, epigenomic, imaging and clinical phenotype data and to infer multilevel omics and image causal networks for the discovery of paths from genetic variants to diseases. The focus of this paper is to survey statistical methods for causal inference and explore the potential to use modern causal inference theory for developing statistics for genome-wide causal studies (GWCS) of complex diseases.

## Causal markov conditions

The widespread view about causation in genetic epidemiology field is that only interventions to a system can discover causal relations. Many epidemiologists and statistical geneticists have doubt about the possibility of using observational data to identify disease-causing variants and “shied away” from causal inference based on observation data (Janzing et al., 2016). In the past decade, great progress in causal inference has been made. In contrast to classical statistics where the relationships between random variables are measured by statistical dependence or association, the algorithms for causal inference that are designed to discover the data generating processes based on statistical observations have been developed.

**Figure 1:**
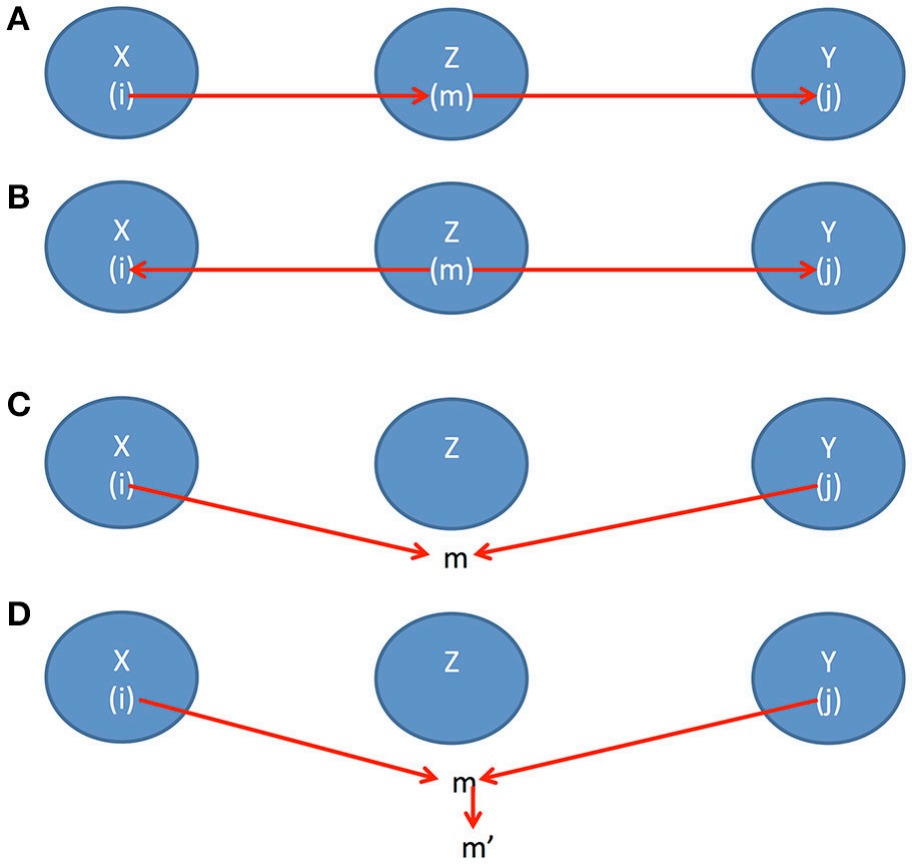
**(A)** Causal direction 1, **(B)** Causal direction 2, **(C)** Causal direction 3, and **(D)** Causal direction 4.

**Figure 2:**
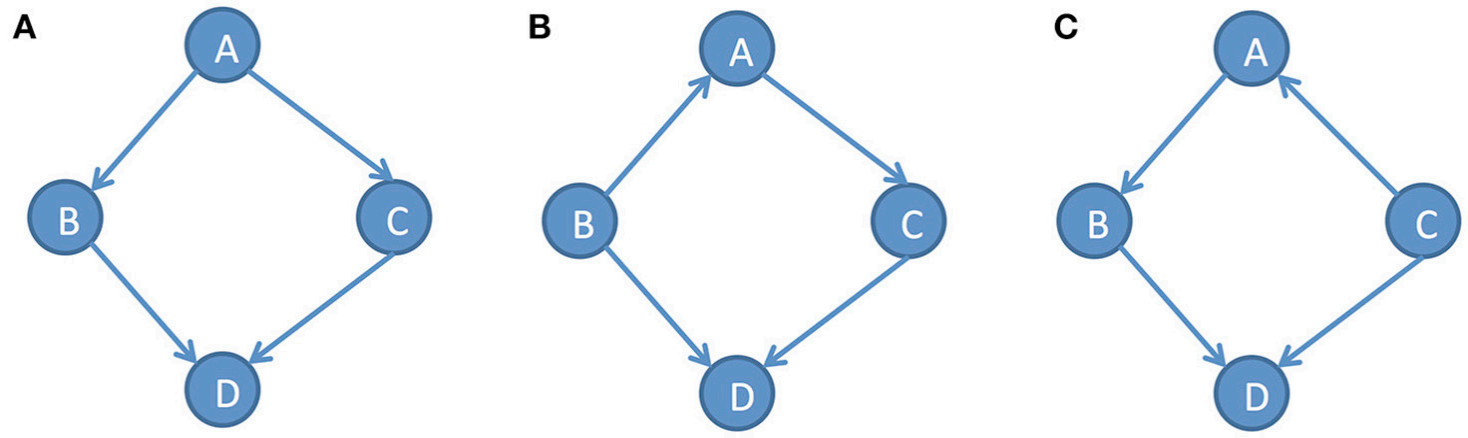
Different DAGs can have same set of conditional independence distributions. **(A)** DAG example A, **(B)** DAG example B, **(C)** DAG example C.

**Figure 3:**
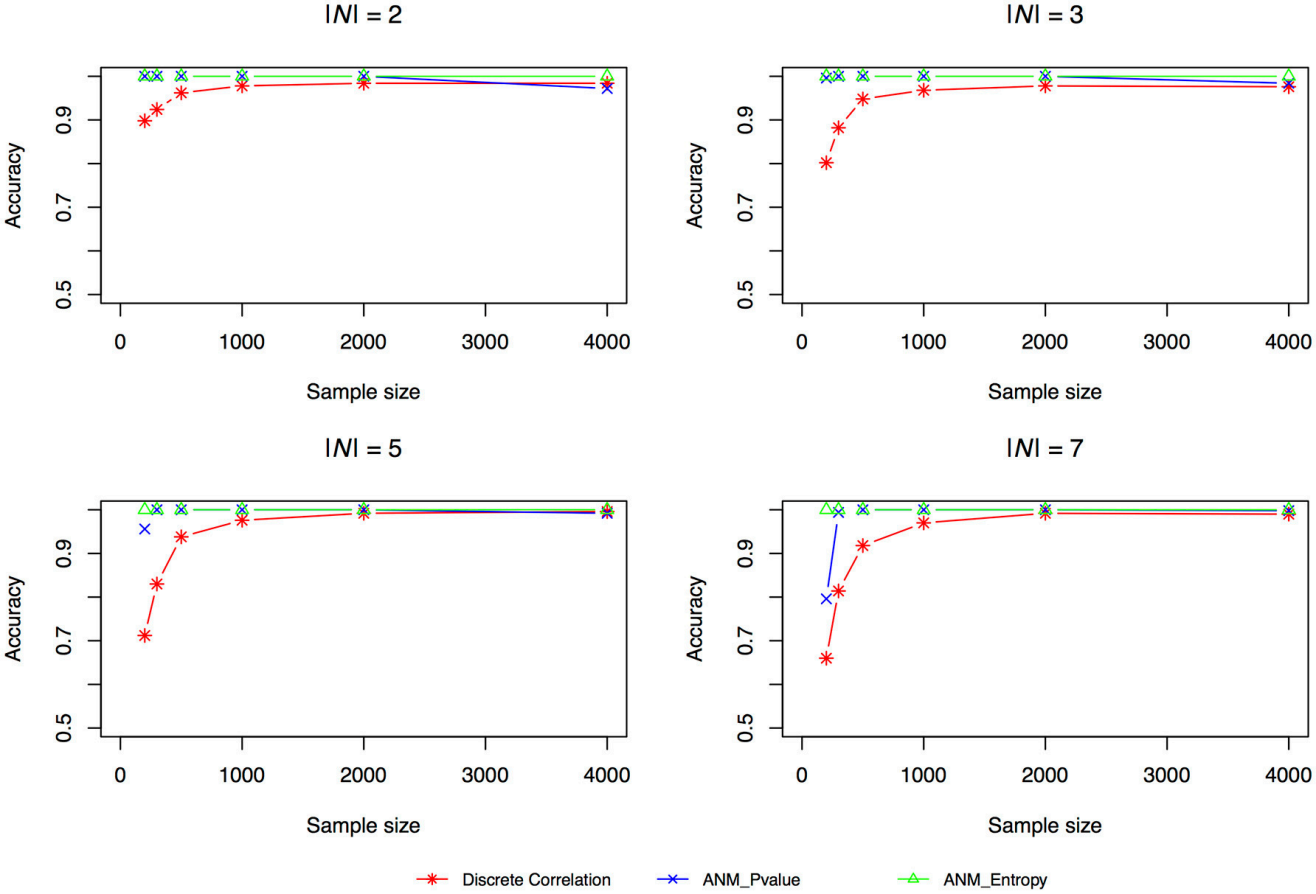
Simulation scenario 1, |N| = 2. Simulation scenario 2, |N| = 3. Simulation scenario 3, |N| = 5. Simulation scenario 4, |N| = 7.

**Figure 4:**
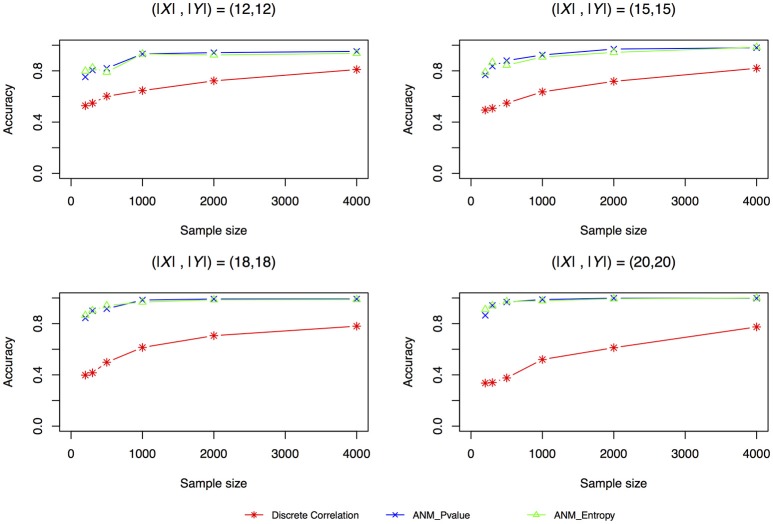
Simulation scenario 1, (|X|,|Y|) = (12,12). Simulation scenario 2, (|X|,|Y|) = (15,15). Simulation scenario 3, (|X|,|Y|) = (18,18). Simulation scenario 4, (|X|,|Y|) = (20,20).

The classical causal inference theory assumes the Causal Markov conditions to infer causal relationships among multiple variables and connect causality with statistics (Janzing and Schölkopf, 2010). Consider a set of variables *V* = {*X*_1_, …, *X*_*n*_}. Let *G* = {*V, E*} be a directed acyclic graph (DAG) where nodes in the DAG represent the random variables *X*_*i*_ and an arrow from node *X*_*i*_ to node *X*_*j*_ denotes a causal direction. Let *P*(*X*_1_, …, *X*_*n*_) be a joint probability distribution of variables *X*_1_, …, *X*_*n*_. The Markov condition describes the causal structure of the DAG *G*. The DAG can also be characterized by the concept of parent sets. Let *pa*_*i*_ and *ND*_*i*_ be the set of parents of the node *X*_*i*_, respectively. The Markov conditions can be formulated as the following three forms (Janzing and Schölkopf, 2010):

1. **Factorization of the joint distribution**:
  The joint distribution *P*(*X*_1_, …, *X*_*n*_) can be factorized into the conditional distribution:
  (1) $$\begin{array}{r} P\left(X_{1},\ldots ,X_{n}\right)=\prod_{i=1}^{n}P\left(X_{i}|pa_{i}\right), \end{array}$$
  where *P*(*X*_*i*_|*pa*_*i*_) is the conditional probability of *X*_*i*_, given the values of all parents of *X*_*i*_.
2. **Local markov condition**:
  Every node is conditionally independent of its non-descendants, given its parents, i.e.,
  $$\begin{array}{l} X_{i}\;⫫ND_{i}|pa_{i}, \end{array}$$
  where *ND*_*i*_ denotes the non-descendent of the node *i*.
3. **Global markov condition**:
  Consider three datasets *X, Y* and *Z*. If *X* and *Y* are d-separated by *Z* then we have
  $$\begin{array}{l} X⫫Y\;|Z. \end{array}$$
  To assess conditional independence in a more general case, we introduce a useful concept, d-separation which associates “correlation” with “connectedness” and independence with “separation”. Two sets of variables *X* and *Y* are d-separated by a third set of variables *Z*if and only if
  1. there is a path *i* → *m* → *j* where *i* ∈ *X, m* ∈ *Z* and *j* ∈ *Y* (Figure 1A) or
  2. there is a path *i* ← *m* → *j* where *i* ∈ *X, m* ∈ *Z* and *j* ∈ *Y* (Figure 1B) or
  3. there is path *i* → *m* ← *j* where *i* ∈ *X, j* ∈ *Y*, but *m* is not in *Z* (Figure 1C) or
  4. there is path *i* → *m* ← *j* where *i* ∈ *X, j* ∈ *Y*, but *m* and its descendants are all not in *Z* (Figure 1D).

In the DAG *G*, we defined the conditional densities *P*(*x*_*i*_|*pa*_*i*_) as the Markov kernels. The set of Markov kernels defines a Markovian density. However, in general, the Markov conditions cannot uniquely determine causal graphs. Many different DAGs satisfy the same set of independence relations. For example, consider three simple DAGs: *x* → *y* → *z*, *x* ← *y* ← *z* and *x* ← *y* → *z*. Three variables *x, y* and *z*in all three DAGs satisfy the same conditional independent distribution: *x* and *z* are independent, given*y*. Figure 2 shows three different DAGs that share the same conditional independent distributions: *B*⫫*C*|*A* and *A*⫫*D*|(*B, C*). These examples show that multiple graphs may satisfy the Markov conditions. Except for Markov conditions, we need other constraints for learning causal structure.

The faithfulness condition is another constraint that helps to infer causal structure from observational data. The faithfulness condition requires that conditional independences in the distribution correspond to the d-separation in the DAG one by one. In other words, faithfulness condition requires that every conditional independence in the distribution must correspond to the Markov condition that is applied to the DAG (Peters et al., 2017; Xiong, 2018).

## Algorithmic complexity for causal inference

Markov condition approaches to causal inference have two serious limitations. First, the approaches based on Markov condition and faithfulness assumption can only identify the graph up to its Markov equivalence class (Janzing and Schölkopf, 2010). Second, the Markov condition approaches need sampling. However, some cases require causal inference for single observation where the Markov condition approaches cannot be applied.

To overcome these limitations, we need to develop methods for causal inference without resorting to probability theory. One approach is to infer causation via algorithmic information theory. We begin with introduction of algorithmic information theory. Consider two 20-bit binary strings:

$$\begin{array}{l} \text{S}=10101010101010101010\\ \text{S}=00011101001000101101, \end{array}$$

which are equally likely to represent the results of 20 flips of coin. However these two strings have large difference in the complexity between them. The first string has a short description: a 20-bit string with 1 in position in odd number, or can be described as “10 10 times,” which consisting of 11 characters. The second string has no obvious simple description. Therefore, the length of the shortest description of the first and second strings are 11 and 20, respectively.

For any string *x*, we define the Kolmogorov complexity *K*(*x*) as the length of the shortest program that generates *x*, denoted as x^*^, using universal prefix Turing machine (Janzing and Schölkopf, 2010). The conditional Kolmogorov complexity *K*(*x*|*y*) of a string *x* given another string *y* is defined as the length of the shortest program that can generate *x* from *y*. The joint Kolmogorov complexity *K*(*x, y*) is defined as the complexity of concatenation *x*′*y* where *x*′ denotes a prefix code of *x*. Mutual information and Markov conditions can be extended to algorithmic mutual information and Algorithmic Markov conditions (Supplementary Note A).

## Additive noise models

In the previous section, we introduce the Markov conditions and faithfulness, which are the constrained-based approaches. These constrained-based approaches cannot identify the unique causal solution or make causal inference between two variables. However, the genome wide association studies (GWAS) test the association of single SNP with the disease. GWAS investigates dependence between two variables. Similar to GWAS, the genome-wide causation studies (GWCS) needs to discover the SNP that causes disease. We need to develop statistical methods for bivariate causal discovery. The DAG approach requires at least variables for causal inference. Therefore, the classical Bayesian networks and DAG-based causal inference methods cannot be applied to the GWCS. To overcome these limitations, some authors (Kano and Shimizu, 2003; Shimizu et al., 2006; Peters et al., 2011) proposed additive noise models (ANMs):

(2) $$\begin{array}{r} Y=f\left(X\right)+N,\;N\;⫫\;X, \end{array}$$

where *f* is a function, and *N* is noise that is independent of the cause *X*. It is clear that the conditional distribution *P*(*Y*|*X*) of the response, given *X* is equal to the noise distribution *P*_*N*_(*Y* − *f*(*X*)). If *X* → *Y* then we should have (Janzing and Steudel, 2010)

(3) $$I(P\left(X\right):P_{N}\left(Y-f\left(X\right)\right)=0.$$

Consider two ANMs: integer models and cyclic models (Peters et al., 2011).

### Integer models

Consider two random variables *X* and *Y* that take integer values (ℤ). The support can be either infinite or finite. Consider an ANM from → *Y*:

(4) $$\begin{array}{r} Y=f\left(X\right)+N,\;N\;⫫\;X, \end{array}$$

where *f* is a function *f*:ℤ → ℤ and *N* is a noise variable. Let *n*(*l*) = *P*(*N* = *l*). In (4), we further assume *n*(0) ≥ *n*(*j*) for all *j* ≠ 0. If there is also an ANM to fit the data, then the ANM is referred to as reversible.

### Cyclic models

We first define the ring ℤ/*mℤ* as the set of remainders modulo *m*, i.e., ℤ/*mℤ* = {[0], [1], …, [*m* − 1]}.

Now we consider random variables that can take values in a periodic domain. Formally, we define *m*-cyclic random variable as the variable that takes values in ℤ/*mℤ*. Let *X* and *Y* be *m* and *k*-cyclic random variables, respectively. Define an ANM from *X* to *Y*:

(5) $$\begin{array}{r} Y=f\left(X\right)+N,\;N\;⫫\;X, \end{array}$$

where *f* is a function *f*:ℤ/*mℤ* → ℤ/*kℤ*, *N* is a *k*-cyclic noise. We assume *n*(0) ≥ *n*(*j*) for all *j* ≠ 0.

Similarly, we can define an ANM from *Y* to :

(6) $$\begin{array}{r} X=g\left(Y\right)+N_{x},\;N_{x}\;⫫\;Y, \end{array}$$

where *g*:ℤ/*kℤ* → ℤ/*mℤ* and *N*_*x*_ is a *m*-cyclic noise.

If both the ANM from *X* → *Y* and ANM from *Y* to *X* hold then the ANM is called reversible. The selection of the integer ANM or cyclic ANM mainly depends on the target *Y* domain.

The cyclic models can be used for genetic studies of complex disease. Let *Y* be a binary trait to indicate the disease status of the individual. Thus, *Y* is a 2-cyclic variable. Thepotential cause variable *X* denotes the genotype of the individual. Thus, *X* is a 3-cyclic variable.

## Identifiability

In most cases, nature selects one causal direction. The causal inference principle states that if *Y* satisfies the ANM from *X* to *Y*, but does not satisfy the ANM from *Y* to *X*, then we infer *X* to be the cause for *Y*, which is denoted by *X* → *Y*. In some cases, *Y* satisfies ANMs in two directions and hence causation does not exist. The question is under what conditions a joint distribution admits an ANM in at most one direction.

The causal inference principle implies that if *X* → *Y*, then the conditional probability *P*(*Y*|*X*) does not depend on the cause *X*. Several examples to illustrate identifiability are given in Supplementary Note B.

## Genetic association analysis and causation analysis

It is well known that correlation (association) and causation are different concepts. Correlation does not imply causation and conversely, causation also does not imply correlation. Our experiences demonstrated that large proportions of causal loci cannot be discovered by association analysis. This explains why a large number of genetic variants still remain hidden. The observed association may be in part due to chance, bias and confounding. Furthermore, a recent study found that “association signals tend to be spread across most of the genome,” by the debatable omnigenic model, which again shows that association signals provide limited information on causes of disease, calling the future of the GWAS into question (Boyle et al., 2017; Callaway, 2017). An observed association may not lead to inferring a causal relationship and the lack of association may not be necessary to imply the absence of a causal relationship. Finding causal SNPs only via searching the set of associated SNPs may not be an effective research direction in genetics. The dominant use of association analysis for genetic studies of complex diseases hampers the theoretical development of genomic science and its application in practice. The examples showing that association and causation are difference concepts are given in Supplementary Note C.

**Algorithm 1:**
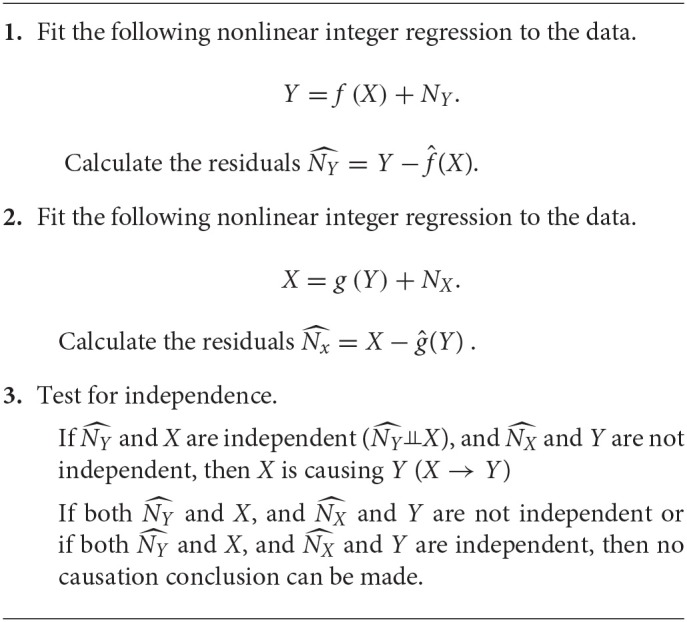
for Causal Genetic Analysis Using ANM:

## Causal genetic analysis

The ANMs can be used for casual genetic analysis of complex disease. The procedures are summarized as follows Peters et al. (2011).

The ordinary egression usually minimizes the sum of square of errors. However, here we evaluate the proposed nonlinear function by checking the independence of regressor and the residuals. Therefore, Peters et al. (2011) suggested using a dependence measure (DM) between regressor and residuals as a lost function. If we simultaneously consider multiple SNPs, the ANMs with a variate (SNP) can be extended to the ANMs with multivariate variables (multiple SNPs). We assume that *W* is a *q* dimensional vector of variables. An ANM with multiple SNPs for causal genetic analysis is given as follows:

(7) $$\begin{array}{r} Y=f\left(W\right)+N,\;W\;⫫\;N, \end{array}$$

where *W* = [*W*_1_, …, *W*_*q*_].

Algorithm 1 in Peters et al. (2011) can be adopted to the regression with multiple regressor. The following algorithm for discrete regression with multiple regressor takes the discrete regression with one regressor as its special case.

where ε and *T* are pre-specified.

For inferring causation involving one vector of variables, distance correlation will be used as *DM*. The problem for testing the independence between cause (regressor) and residuals can be formulated as a 2 × *q* contingence table (Table 1). Let *n*_0_ and *n*_1_ be number of individuals with *N* = 0 and *N* = 1, respectively. Let *n* = *n*_0_ + *n*_1_. Let *g*_*j*_1…_*j*_*q*__ denote the genotype of *q* SNPs, *a*_*j*_1…_*j*_*q*__ and *b*_*j*_1…_*j*_*q*__ be the number of individuals with genotype *g*_*j*_1…_*j*_*q*__, and *N* = 0 and *N* = 1, respectively. Define the marginal frequencies: $\frac{n_{0}}{n},\;\frac{n_{1}}{n}$ and $\frac{a_{j_{1\ldots }j_{q}}{+b}_{j_{1\ldots }j_{q}}}{n}$. Then, we obtain

**Table 1:** Contingency table for testing independence.

	****Genotype****	****Genotype****	**g_j_1 …_j_q__**	****Genotype****	****Genotype****
	**g_1 1 … 1_**	**…**		**…**	**g_3.3 … 3_**	
*N* = 0	*a*_1 1… 1_	…	*a*_*j*_1…_*j*_*q*__	…	*a*_3.3… 3_	*n*_0_
*N* = 1	*b*_1 1… 1_	…	*b*_*j*_1…_*j*_*q*__	…	*b*_3.3… 3_	*n*_1_
	*a*_1 1… 1_	…	*a*_*j*_1…_*j*_*q*__+	…	*a*_3.3… 3_+	*n* = *n*_0_ + *n*_1_
	+*b*_1 1… 1_		*b*_*j*_1…_*j*_*q*__		*b*_3.3… 3_	

**Algorithm 2:**
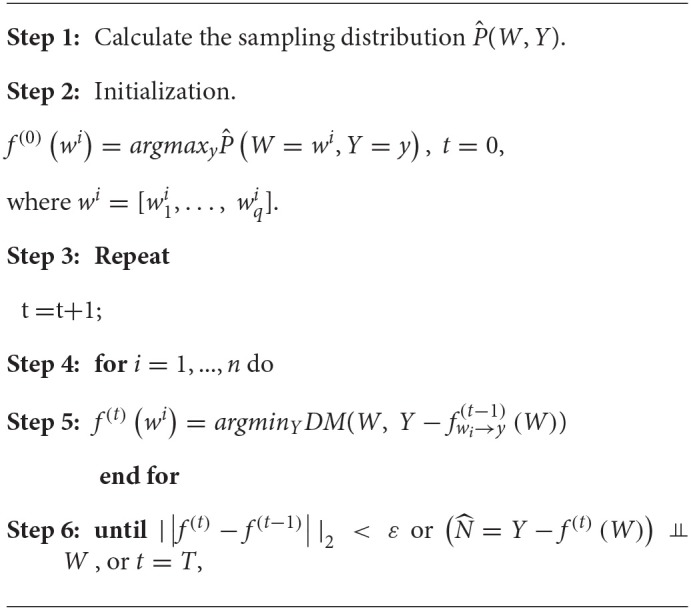
(Distance Regression With Dependence Measure)

$E[a_{j_{1\ldots }j_{q}}]=\frac{1}{n}n_{0}(a_{j_{1\ldots }j_{q}}+b_{j_{1\ldots }j_{q}})$ and $\text{E}[b_{j_{1\ldots }j_{q}}]=\frac{n_{1}}{n}(a_{j_{1\ldots }j_{q}}+b_{j_{1\ldots }j_{q}})$. Then, the test statistic for testing independence is defined as

(8) $$T=\sum_{j_{1}j_{2}\ldots j_{q}}\left[\text{​}\frac{{\left({\hat{a}}_{j_{1\ldots j_{q}}}-E[a_{j_{1\ldots }j_{q}}]\right)}^{2}}{E[a_{j_{1\ldots }j_{q}}]}+\frac{{\left({\hat{b}}_{j_{1}\ldots j_{q}}-E[b_{j_{1\ldots }j_{q}}]\right)}^{2}}{E[b_{j_{1\ldots }j_{q}}]}\text{​}\right],$$

where â_*j*_1…_*j*__*q*___ and ${\hat{b}}_{j_{1}\ldots j_{q}}$ are observed values of *a*_*j*_1…_*j*_*q*__ and *b*_*j*_1…_*j*_*q*__, respectively. Under the null hypothesis of independence, the test statistic *T* is distributed as a central $\chi_{\left(3^{q}-1\right)}^{2}$ distribution with degrees of freedom 3^*q*^−1.

If SNPs involve rare variants or number of SNPs increases, the expected counts of many cells will be small. Fisher's exact test should be used to test for independence.

## Causation identification using entropy

The ANM assumes that noise or any outside factor (exogenous variable) affects the effect variable additively, i.e.,

$$\begin{array}{l} Y=f\left(X\right)+E,\;E\;⫫\;X. \end{array}$$

The ANM can be extended to more general nonlinear model (Kocaoglu et al., 2016). We assume that variable *Y* is an arbitrary function of *X* and an exogenous variable *E*:

(9) $$\begin{array}{r} Y=f\left(X,E\right),\;X\;⫫\;E, \end{array}$$

where we assume that the exogenous variable *E* has low Renyi entropy. Renyi entropy is defined as

(10) $$\begin{array}{r} H_{0}\left(E\right)=\log k, \end{array}$$

where *k* is the number of states of the variable *E*.

In other words, if the model in the wrong causal direction: *X* = *g*(*Y*, Ẽ), then the exogenous variable Ẽ has higher Renyi entropy than that of *E*, i.e., *H*_0_(*E*) ≤ *H*_0_(Ẽ).

Since Renyi entropy *H*_0_ is difficult to compute, we can replace Renyi entropy by Shannon entropy *H*_1_. An algorithm for inferring the true causal direction is to find the exogenous variable *E* with the smallest *H*_1_(*E*) such that

$$\begin{array}{l} Y=f\left(X,E\right),\;X\;⫫\;E. \end{array}$$

Kocaoglu et al. (2016) proposed an algorithm to calculate the Sharon entropy *H*_1_(*E*). They make argument that

(11) $$\begin{array}{r} P\left(Y=y|X=x\right)=P\left(f\left(x,E\right)=y\right). \end{array}$$

If we define *f*_*x*_(*E*) = *f*(*x, E*), then it follows from Equation (11) that we can use the conditional distribution *P*(*Y*|*X*) to calculate the distribution of *E*: *P*(*Y* = *y*|*X* = *x*) = *P*(*f*_*X*_(*E*) = *y*). Algorithm 1 in Kocaoglu et al. (2016) assumed that the number of states of the variables *X* and *Y* is equal, which prevents application of Algorithm 1 to causal genetic analysis for genotype data. However, if we consider allele distribution data, Algorithm 1 in Kocaoglu et al. (2016) can still be applied to the causal genetic analysis. Consider the matrix

$$M=\left[\begin{array}{cc} P_{y}(0) & P_{x}(0)\\ P_{y}(1) & P_{x}(1) \end{array}\right],$$

where

$$x=\{\begin{array}{cc} 0 & a\\ 1 & A \end{array}\;,\;y=\{\begin{array}{cc} 0 & Normal\\ 1 & disease \end{array}\;.$$

Algorithm 1 in Kocaoglu et al. (2016) can then be written as follows.

Step 1. Input matrix *M* = (*P*_*i*_(*j*))_2 × 2_.
Step 2. Define *e* = [⊢].
Step 3. Sort each row of *M* in decreasing order. $p_{i}\left(1\right)=\underset{j}{\max}\left\{p_{i}\left(j\right)\right\}$.
Step 4. Search minimum of maximum of each row: $\alpha \;\underset{i}{\min}\left(p_{i}\left(1\right)\right)$.
Step 5. While α > 0 do
  *e* ← [*e*, α],
  Remove α from maximum of each row: *p*_*i*_(1) *p*_*i*_(1) − α, for all *i*
  Sort each row of updated *M* in decreasing order.
  $\alpha \leftarrow \underset{i}{\min}\left\{P_{i}\left(1\right)\right\}$.
Step 6. End while
Step 7. Output *e*.

For the genotype data, we suggest to use Sharron entropy *H*_1_(*E*) and *H*_1_(Ẽ) as a DM in the ANM and compare *H*_1_(*E*) with *H*_1_(Ẽ). If *H*_1_(*E*) < *H*_1_(Ẽ) then *X* → *Y*; if *H*_1_(Ẽ) < *H*_1_(*E*) then *Y* → *X*.

## Distance correlation for causal inference with discrete variables

In previous sections, we introduce the basis principal for assessing causation *X* → *Y* that the distribution *P*(*X*) of causal *X* is independent of the causal mechanism or conditional distribution *P*(*Y*|*X*) of the effect *Y*, given causal *X*. Now the question is how to assess their independence. Recently, distance correlation is proposed to measure dependence between random vectors which allows for both linear and nonlinear dependence (Sze'kely et al., 2007; Sze'kely and Rizzo, 2009). Distance correlation extends the traditional Pearson correlation in two remarkable directions:

1. Distance correlation extends the Pearson correlation defined between two random variables to the correlation between two sets of variables with arbitrary numbers;
2. Zero of distance correlation indicates independence of two random vectors.

Discretizing distributions *P*(*X*) and *P*(*Y*|*X*), and viewing their discretized distributions as two vectors *P*(*X*) and *P*(*Y*|*X*), the distance correlation between *P*(*X*) and *P*(*Y*|*X*) can be used to assess causation between *X* and *Y*.

Consider two vectors of random variables: *p*-dimensional vector *X* and *q*-dimensional vector*Y*. Let *P*(*x*) and *P*(*y*) be density functions of the vectors *X* and *Y*, respectively. Let *P*(*x, y*) be the joint density function of *X* and *Y*. There are two ways to define independence between two vectors of variables: (1) density definition and (2) characteristic function definition. In other words, if *X* and *Y* are independent then either

1. *P*(*x, y*) = *P*(*x*)*P*(*y*) or
2. *f*_*X, Y*_(*t, s*) = *f*_*X*_(*t*)*f*_*Y*_(*s*),

where $f_{X,Y}\left(t,s\right)=E\left[e^{i\left(t^{T}x+s^{T}y\right)}\right]$, $f_{X}\left(t\right)=E\left[e^{it^{T}x}\right]$ and $f_{Y}\left(s\right)=E\left[e^{is^{T}y}\right]$ are the characteristic functions of (*X, Y*), *X* and *Y*, respectively. Therefore, we can use both distances ||*P*(*x, y*) − *P*(*x*)*P*(*y*)|| and ||*f*_*X, Y*_(*t, s*) − *f*_*X*_(*t*)*f*_*Y*_(*s*)|| to measure dependence between two vectors *X* and *Y*. Distance correlation (Sze'kely et al., 2007) uses distance between characteristic function to define the dependence measure.

Assume that pairs of (*X*_*k*_, *Y*_*K*_), *k* = 1, …, *n* are sampled. Calculate the Euclidean distances:

$$\begin{array}{l} a_{kl}=||X_{K}-X_{l}|{|}_{p},b_{kl}=||Y_{k}-Y_{l}|{|}_{q},k=1,\ldots ,n,l=1,\ldots ,n. \end{array}$$

Define

$$\begin{array}{l} {ā}_{k.}=\frac{1}{n}\sum_{l=1}^{n}a_{kl},\;{ā}_{.l}=\frac{1}{n}\sum_{k=1}^{n}a_{kl},\\ {ā}_{..}=\frac{1}{n^{2}}\sum_{k=1}^{n}\sum_{l=1}^{n}a_{kl}, \end{array}$$

$$\begin{array}{l} {\bar{b}}_{k.}=\frac{1}{n}\sum_{l=1}^{n}b_{kl},\;{\bar{b}}_{.l}=\frac{1}{n}\sum_{k=1}^{n}b_{kl}\text{ and}\\ {\bar{b}}_{..}=\frac{1}{n^{2}}\sum_{k=1}^{n}\sum_{l=1}^{n}b_{kl}. \end{array}$$

Define two matrices:

$$\begin{array}{l} A={\left(A_{kl}\right)}_{n\times n}\text{ and }B={\left(B_{kl}\right)}_{n\times n}, \end{array}$$

where

$$\begin{array}{l} A_{kl}=a_{kl}-{ā}_{k.}-{ā}_{.l}+{ā}_{..},\\ B_{kl}=b_{kl}-{\bar{b}}_{k.}-{\bar{b}}_{.l}+{\bar{b}}_{..},\;k,l=1,\ldots ,n. \end{array}$$

Finally, the sampling distance covariance*V*_*n*_(*X, Y*), variance *V*_*n*_(*X*) and correlation *R*_*n*_(*X, Y*) are defined as

(12) $$\begin{array}{r} V_{n}^{2}\left(X,Y\right)=\frac{1}{n^{2}}\sum_{k=1}^{n}\sum_{l=1}^{n}A_{kl}B_{kl}, \end{array}$$

$$\begin{array}{l} V_{n}^{2}\left(X\right)=V_{n}^{2}\left(X,X\right)=\frac{1}{n^{2}}\sum_{k=1}^{n}\sum_{l=1}^{n}A_{kl}^{2},\\ V_{n}^{2}\left(Y\right)=\sum_{k=1}^{n}\sum_{l=1}^{n}B_{kl}^{2}, \end{array}$$

(13) $$R_{n}^{2}(X,Y)=\{\begin{array}{cc} \frac{V_{n}^{2}(X,Y)}{\sqrt{V_{n}^{2}(X)V_{n}^{2}(Y)}}, & V_{n}^{2}(X)V_{n}^{2}(Y)>0\\ 0 & V_{n}^{2}(X)V_{n}^{2}(Y)=0, \end{array}$$

respectively.

Independence can be formally tested by statistics based on distance correlation. The null hypothesis is defined as *H*_0_:*X* and *Y* are independent.

We can use Equation (12) to define a test statistic:

(14) $$\begin{array}{r} T_{IND}=n\frac{V_{n}^{2}\left(X,Y\right)}{{ā}_{..}{\bar{b}}_{..}}. \end{array}$$

Since distribution of test statistics is difficult to compute, we often use permutations to calculate *P*-values. Specifically, we can permutate *X* and *Y*millions of times. For each permutation, we compute test statistic *T*_*IND*_. Therefore, via permutations we can calculate the empirical distribution of *T*_*IND*_. Using an empirical distribution, we can calculate the *P*-value as

$$\begin{array}{l} P-value=P\left(T_{IND}>T_{IND0}\right), \end{array}$$

where *T*_*IND*0_ is the observed value of *T*_*IND*_ in real data.

Distance correlation can be used to test independence between causal and causal generating mechanisms (Liu and Chan, 2016). Consider *p* -dimensional random vector*X* and *q* -dimensional random vector*Y*. Let *P*(*X, Y*) be their joint distribution. Let *P*(*X*) and *P*(*Y*|*X*) be the density function of *X* and conditional density function of *Y*, given*X*, respectively. Similarly, we can define *P*(*Y*) and *P*(*X*|*Y*). Unlike association analysis where dependence is measured between two random vectors, in causal analysis, dependence is measured between two distributions.

The distance correlation dependence measures between two distributions are defined as

(15) $$\begin{array}{r} \Delta_{X\to Y}=R\left(P\left(X\right),P\left(Y|X\right)\right), \end{array}$$

(16) $$\begin{array}{r} \Delta_{Y\to X}=R\left(P\left(Y\right),P\left(X|Y\right)\right), \end{array}$$

where *R*(., .) is a distance correlation measure between two vectors.

Suppose that *X* and *Y* are discretized (or divided) into *m* and *k* groups, respectively. Let *m*_*i*_ be the number of points *X* in the *ith* group and *k*_*ij*_ be the number of points (*X, Y*) where *X* is in the *ith* group and *Y* is in the *jth* group. Then, $n=\sum_{i=1}^{m}m_{i}$ and $m_{i}=\sum_{j=1}^{k}k_{ij}$. Let *X*^(*i*)^ be the collection of all points *X* in the *ith* group and *Y*^(*j*)^ be the collection of all points *Y* in the *jth* group. Then, the estimated density function *P*(*X*^(*i*)^) is $P\left(X^{\left(i\right)}\right)=\frac{m_{i}}{n}$ and the conditional density function $P\left(Y^{\left(j\right)}|X^{\left(i\right)}\right)=\frac{k_{ij}}{m_{i}}$.

Let *S*_*X* → *Y*_ = *a*_.._*b*_.._. Distance covariance is defined as

(17) $$\begin{array}{r} V_{m}^{2}\left(P\left(X\right),P\left(Y|X\right)\right)=\frac{1}{m^{2}}\sum_{i=}^{m}\sum_{j=1}^{m}A_{ij}B_{ij}. \end{array}$$

Similarly, $V_{m}^{2}\left(P\left(Y\right),P\left(X|Y\right)\right)$ and *S*_*Y* → *X*_ can be similarly defined.

Define

(18) $$\begin{array}{r} \Delta_{X\to Y}=\frac{mV_{m}^{2}\left(P\left(X\right),P\left(Y|X\right)\right)}{S_{X\to Y}}, \end{array}$$

(19) $$\begin{array}{r} \Delta_{Y\to X}=\frac{mV_{m}^{2}\left(P\left(Y\right),P\left(X|Y\right)\right)}{S_{Y\to X}}. \end{array}$$

The null hypothesis for testing is

*H*_0_: no causation between two vectors *X* and *Y*.

The statistic for testing the causation between two vectors *X* and *Y* is defined as

(20) $$\begin{array}{r} T_{C}=|\Delta_{X\to Y}-\Delta_{Y\to X}|. \end{array}$$

When *T*_*C*_ is large, either Δ_*X*→ *Y*_ > Δ_*Y*→ *X*_ which implies *Y*causes *X*, or Δ_*Y*→ *X*_ > Δ_*X*→ *Y*_ which implies that *X* causes *Y*. When *T*_*C*_ ≈ 0, this indicates that no causal decision can be made.

## Simulations

We use simulation experiments that were presented in Liu and Chan (2016) to compare the performance of three methods: ANM, Distance correlation and entropy for causal inference with discrete variables. Consider two sets of data (1) dataset 1 and dataset 2 generated in section (Additive Noise Models) and section (Models with Randomly Generated P(X) and P(Y|X)) of the paper written by (Liu and Chan, 2016), respectively.

The accuracies of three methods for causation discovery in dataset 1 were shown in Figure 3. A total of 200, 300, 500, 1,000, 2,000, and 4,000 points for each model were sampled. Figure 3 showed that the Entropy-based ANMs where the independence between cause *X* and residuals *E* are tested by entropy had the highest accuracies to infer cause-effect direction, the distance-correlation-based methods had the lowest accuracies, and the classical ANMs with discrete variables had the similar accuracies as that the entropy-based ANMs had. We observed that as sample sizes increased the accuracies increased and when the sample sizes are larger than 2,000 all three methods can accurately infer cause-effect directions.

Figure 4 plotted accuracy of three methods for inferring cause-effect direction as a function of sample sizes. Unlike the results in Liu and Chan (2016) where they showed that distance correlation had much higher accuracy to infer causal direction than the ANMs, we observed in Figure 4 that both entropy-based ANMs and classical ANMs had similar accuracies and had higher accuracies than the distance correlation method. We also observed that even when sample sizes reached 4,000 the distance correlation method still could not reach accuracy beyond 80%.

To further evaluate their performance, we calculated the type I error rates three statistics for testing causation using simulations. We randomly selected 10 SNPs across the genome from 1,000 Genome Project data. A total of 1,000 simulations were conducted. We consider two scenarios: (1) no association and no causation and (2) presence of association, but no causation. Tables 2, 3 presented average type 1 error rates of three tests over 10 SNPs. Tables 2, 3 showed that type 1 error rates of the ANM based on permutation and DC method even in the presence of association were not significantly deviated from nominal levels, but the type 1 error rates of ANM based on entropy under association were significantly deviated from the Nominal levels. These results showed that the ANM with permutation tests and DC methods can be used for testing causation between SNP and disease, but the ANM based on entropy cannot be applied to test causation between SNP and disease.

**Table 2:** Average type 1 error rates of three statistics for testing causation, assuming no association.

	**Type I error rate**			
**Methods**	**α**	***N* = 500**	***N* = 1,000**	***N* = 2,000**
ANM Permutation	0.05	0.0431	0.0504	0.0505
	0.01	0.0090	0.0073	0.0081
ANM Entropy	0.05	0.0390	0.0392	0.0387
	0.01	0.0093	0.0080	0.0078
DC	0.05	0.0470	0.0461	0.0457
	0.01	0.0074	0.0091	0.0097

**Table 3:** Average type 1 error rates of three statistics for testing causation, assuming presence of association.

	**Type I error rate**			
**Methods**	**α**	***N* = 500**	***N* = 1,000**	***N* = 2,000**
ANM Permutation	0.05	0.0341	0.0355	0.0337
	0.01	0.0094	0.0117	0.0103
ANM Entropy	0.05	0.1962	0.2014	0.2169
	0.01	0.1666	0.1679	0.1705
DC	0.05	0.0507	0.0511	0.0508
	0.01	0.0103	0.0093	0.0099

To give some recommendations on when and which methods should be used, we conducted power simulations using the data for type 1 error calculation. We assume that both association and causation exist as described in Supplementary Note C. The results were summarized in Table 4. Table 4 showed that in all scenarios, the ANM-based on permutation had the highest power among three statistical methods for testing causation.

**Table 4:** Power of three statistical methods for testing causation in the presence of both association and causation.

		**Number of samples**				
		**200**	**500**	**1,000**	**2,000**	**5,000**
ANM *P*-value	0.05	0.5731	0.6983	0.7642	0.8127	0.8466
	0.01	0.5193	0.6830	0.7654	0.8239	0.8783
ANM Entropy	0.05	0.1290	0.1496	0.1611	0.1686	0.1747
	0.01	0.1120	0.1308	0.1470	0.1555	0.1771
DC	0.05	0.2549	0.3104	0.3458	0.3834	0.4175
	0.01	0.0954	0.1214	0.1476	0.1840	0.2400

## Real data analysis

Illustrate the application of causal inference to genetic analysis of complex diseases, three methods for causal inference were used to infer causal relationships between four SNPs in two genes with Alzheimer's disease (AD). Two SNPs in genomic positions 15528889 and 15530350 in gene *TRIM16,* two SNPs 15524749 and 15519576 in gene *CDRT1* and other two SNPs were types in 1,707 individuals (514 AD and 1,193 controls). The results were summarized in Table 5. Due to limitation of computer capability, only 1,000,000 permutations were carried out to compute *P*-values of classical ANM test statistics and entropy-based ANM test statistics. The threshold for declaring significance of association test was 1.14 × 10^−8^. Since distance correlation test requires that the potential cause should take more than three values of states, in general, it cannot be used for causal genetic analysis. Table 5 showed that *P*-values of the classical ANM and entropy-based ANM were close, and that four SNPs in two genes which showed strong association also demonstrated causation. The literature reported that gene *TRIM16* inhibited neuroblastoma cell proliferation through cell cycle regulation and dynamic nuclear localization and gene *CDRT1* was involved in frontotemporal dementia (Aronsson et al., 1998; Bell et al., 2013).

**Table 5:** *P*-values for testing the causation and association of four SNPs with AD.

			***P*****-values**			
**Chr**	**Gene**	**Genomic position**	**Association**	**ANM**	**ANM-Entropy**	**Distance correlation**
17	TRIM16	15528889	4.51E-09	<1.00E-06	<1.00E-06	0.5
17	TRIM16	15530350	1.01E-07	<1.00E-06	<1.00E-06	0.5
17	CDRT1	15524749	5.12E-08	<1.00E-06	<1.00E-06	0.5
17	CDRT1	15519576	5.49E-09	<1.00E-06	<1.00E-06	0.5
1	PYHIN1	158947655	0.00131	0.00011	0.00031	0.15831
4	AFAP1	7813044	0.00222	0.00083	0.00074	0.58278

## Future perspective

Association analysis has been used as a major tool for dissecting genetic architecture and unraveling mechanisms of complex diseases for more than a century (Fisher, 1918; Timpson et al., 2017). Although significant progress in dissecting the genetic architecture of complex diseases by genome-wide association studies (GWAS) has been made, the overall contribution of the new identified genetic variants to the diseases is small and a large fraction of disease risk genetic variants is still hidden. Understanding the etiology and causal chain of mechanism underlying many complex diseases remains elusive. The current approach to uncovering hidden genetic variants is (1) to increase sample sizes, (2) to study association of rare variants by next-generation sequencing and (3) to perform multi-omic analysis. Association and correlation analysis are the current paradigm of analysis for all these approaches. Our experiences in association analysis strongly demonstrate that association analysis lacks power to discover the mechanisms of the diseases for the two major reasons. The first reason is that association analysis cannot identify causal signals that are quite different from the association signals. The second reason is that the widespread networks that are constructed in integrated omic analysis are undirected graphs. Using undirected graphs, we are unable to infer direct cause-effect relations and hence cannot discover chain of causal mechanism from genetic variation to diseases via gene expressions, epigenetic variation, protein expressions, metabolism variation, and phenotype variations. The use of association analysis as a major analytical platform for genetic studies of complex diseases is a key issue that hampers the theoretical development of genomic science and its application in practice. Causal inference coupled with multiple omics, imaging, physiological and phenotypic data is an essential component for the discovery of disease mechanisms. It is time to develop a new generation of genetic analysis for shifting the current paradigm of genetic analysis from shallow association analysis to deep causal inference. This review paper introduced major statistical methods for inferring causal relationships between discrete variables and explored the potential roles causal inference may play in genetic analysis of complex diseases. Our purpose is to stimulate discussion about what research direction in genetic studies: causal analysis or association analysis should be taken in the future.

## Author contributions

PH perform data analysis, write paper. RJ perform data analysis; LJ design project. MX design project and write paper.

### Conflict of interest statement

The authors declare that the research was conducted in the absence of any commercial or financial relationships that could be construed as a potential conflict of interest.

## Footnotes

^1^A catalog of Published Genome-Wide Association Studies. 2017. Available from: https://www.genome.gov/page.cfm?pageid=26525384&clearquery=1#result_table
