# Supplementary material for: Application of Causal Inference to Genomic Analysis: Advances in Methodology
Source: Front Genet. 2018 Jul 10;9:238. doi: 10.3389/fgene.2018.00238 (PMC6048229; doi:10.3389/fgene.2018.00238)
Supplement: Supplementary file 1 [file Presentation_1.PDF]

Supplementary note A

Algorithmic Mutual Information and Algorithmic Markov Conditions

Algorithmic mutual information between two strings x and y is defined as

$$I(x{:}\,y) = K(y) - K(y|x^*), \tag{S1}$$

which can also be defined as

$$I(x{:}\,y) = K(x) + K(y) - K(x, y). \tag{S2}$$

Recall that for every string x, x^* is defined as the shortest description. Knowing x^* is equivalent to knowing the pair $(x, K(x))$. Therefore, equation (S1) can be rewritten as

$$I(x{:}\,y) = K(y) - K(y|x, K(x)). \tag{S3}$$

The mutual information can be generalized to the conditional algorithmic mutual information.

Let three strings be denoted by x, y and z. The conditional algorithmic mutual information of x , y, given z is defined as

$$I(x{:}\,y|z) = K(y|z) - K(y|x^*, z)$$

$$= K(y|z) - K(y|x, K(x, z), z). \tag{S4}$$

Equation (S4) can also be written as

$$I(x{:}\,y|z) = K(x|z) + K(y|z) - K(x, y|z). \tag{S5}$$

We use the conditional algorithmic mutual information to define algorithmic conditional independence. In other words, if

$$I(x{:}\,y|z) = 0\ . \tag{S6}$$

then x is conditionally independent of y, given z , which is denoted as

$x \perp\!\!\!\perp y|z$.

Equation (S6) indicates that given z, the additional information of y does not provide a stronger compression of y.

To link the algorithmic mutual information with statistical mutual information, we assume that a string is randomly generated (Janzing and Schölkopf 2010). Specifically, suppose that $x = x_1 x_2 \ldots . x_n$ is a string and symbols $x_i \in \boldsymbol{\mathcal{A}}$ are drawn independently and identically from a probability distribution $P(X)$ where $\boldsymbol{\mathcal{A}}$ denotes the final alphabet set. Therefore, $P(X) = P(x_1) \ldots P(x_n)$. Let $H(x)$ be the entropy of a probability distribution. Intuitively, we have

$$\frac{1}{n} E[K(x)] = \frac{1}{n} \sum_{i=1}^{n} K(x_i) \approx \mathrm{H(X)}. \quad \text{(S7)}$$

Similarly, from equation (S7) we can obtain

$$\frac{1}{n} E[I(x: y)] = \frac{1}{n} E[K(x)] + \frac{1}{n} E[K(y)] - \frac{1}{n} E[K(x, y)]$$

$$\approx H(X) + H(Y) - H(X, Y) = I(X; Y). \quad \text{(S8)}$$

Now we generalize Markov condition to the algorithmic Markov condition. Consider a DAG with n nodes $x_1, \ldots, x_n$. We code these n variables by n strings. Let pa_i be the concatenation of all parents of x_i and nd_i the concatenation of all its non-descendants except x_i itself. Then, the algorithmic Markov condition is

$$x_i \perp\!\!\!\perp nd_i | pa_i^*, \quad \text{(S9)}$$

where pa_i^* represents the optimal joint compression of the parent strings.

Now we are ready to extend the equivalence of Markov conditions to the equivalence of algorithmic Markov conditions (Janzing and Schölkopf 2010):

1. **Factorization of the joint complexity.** The joint complexity of the nodes in the DAG is equal to the sum of conditional complexity of each node, given the optimal compression of its parents:

   $K(x_1, \ldots, x_n) = \sum_{i=1}^{n} K(x_i | pa_i^*)$. (S10)

2. **Local algorithmic Markov condition.** Every node is algorithmically independent of its non-descendants, given the optimal compression of its parents:

   $I(x_i : nd_i | pa_i^*)=0$. (S11)

3. **Global algorithmic Markov condition.** Consider three datasets X, Y and Z. If X and Y are d-separated by Z then we have

   $I(X: Y | Z^*) = 0$. (S12)

Consider two random variables: X and Y. If X causes Y $(X \rightarrow Y)$ then the marginal distribution $P(X)$ and the conditional distribution $P(Y|X)$ of Y, given X are independent. In statistics, although dependence between two random variables can be measured, there are no measures to quantify dependence between two distributions. We use algorithmic mutual information to measure dependence between two distributions. Therefore, if $X \rightarrow Y$, then the algorithmic mutual information between the marginal distribution $P(X)$ of the cause X and the conditional distribution $P(Y|X)$ of effect Y, given cause X is equal to zero, i.e.,

$I\big(P(X): P(Y|X)\big) = 0$ (S13)

or

$$K\big(P(X,Y)\big) = K\big(P(X)\big) + K(P(Y|X)). \tag{S14}$$
