# Supplementary material for: Application of Causal Inference to Genomic Analysis: Advances in Methodology
Source: Front Genet. 2018 Jul 10;9:238. doi: 10.3389/fgene.2018.00238 (PMC6048229; doi:10.3389/fgene.2018.00238)
Supplement: Supplementary file 2 [file Presentation_2.PDF]

Supplementary Note B

Examples for Illustrating Indetifiability

Example 1.

Assume the model: $Y = \sqrt{x} + N$, $x = 1, 4, 9$ and $N = 0, 1, 2, 3$, each with probability $\frac{1}{4}$. Figure S1 shows the simulated data where the dots indicate a probability greater than 0. This example demonstrates that the conditional distribution $P(y|x) = P(N) = \frac{1}{4}$ does not depends on x, i.e., the joint distribution $P(x, y)$ allows an ANM from $X \rightarrow Y$. However, $P(x = 1|y = 1) = 1$, $P(x = 1|y = 2) = \frac{1}{2}$, $P(x = 1|y = 3) = \frac{1}{3}$ and $P(x = 1|y = 4) = \frac{1}{3}$ depends on the values of y. This shows that the joint distribution does not allow an ANM from Y to X.

Peters et al. (2011) gave the conditions under which the ANM holds at most one direction. Specifically, consider an ANM from X to Y. Assume that X takes m values $x_1, \dots, x_m$, the noise N takes values from N_{min} to N_{max} and the function f is a nonconstant. Further assume $min_{(i,j)\in\{1,\dots,m\}:i\neq j} f(x_i) - f(x_j) \leq N_{max} - N_{min}$.

Then, the joint distribution does not admit an ANM from Y to X except for a parameter set of measure 0.

For cyclic models, in general, the ANMs are not reversible. However, the number of reversible cases for the cyclic models is larger than that for the integer models (Peters et al. 2011). Now we give the conditions under which the ANMs are reversible, i.e., both the ANMs from X to Y:

$$Y = f(X) + N, \; N \perp\!\!\!\perp X \quad \text{(SB1)}$$

and the ANMs from Y to X:

$$X = g(Y) + \widetilde{N},\ \ \widetilde{N} \perp\!\!\!\perp Y \qquad \text{(SB2)}$$

hold.

Peters et al (2011) showed that the model $Y = f(x)$ +N is reversible if and only if

$$P(x,y) = P(x)P(y|x) = P(x)n\big(y - f(x)\big) = P(y)P(x|y) = q(y)\tilde{n}(x - g(y)), \qquad \text{(SB3)}$$

where

$q(y) = P(Y = y) = \sum_{\tilde{x}} P(\tilde{x}\,, y) = \sum_{\tilde{x}} P(\tilde{x}\,, y) = \sum_{\tilde{x}} P(\tilde{x})P(y|\tilde{x}) = \sum_{\tilde{x}} P(\tilde{x})n(y - f(\tilde{x}))$ and

$\tilde{n}(a) = P(x|\tilde{y}) = \frac{P(x,\tilde{y})}{q(\tilde{y})} = \frac{P(x)P(\tilde{y}|x)}{q(\tilde{y})} = \frac{P(g(\tilde{y})+a)n(\tilde{y}-f(g(\tilde{y})+a))}{q(\tilde{y})}$.

If X and Y are independent then condition (SB4) will be satisfied and the ANMs are reversible. In fact, define the ANMs:

$$X = g(Y) + X \text{ with } g \equiv 0. \qquad \text{(SB5)}$$

By independent assumption, we have

$$P(x,y) = P(X = x)P(Y = y) = P(x)P(y = f(x) + n) = P(x)P\big(n = y - f(x)\big) = P(x)n(y - f(x)). \qquad \text{(SB6)}$$

By independent assumption and model (24), we obtain

$$P(x,y) = P(Y = y)P(X = x) = q(y)P\big(\widetilde{N} = x\big) = q(y)\tilde{n}(x) = q(y)\tilde{n}(x - g(y)). \qquad \text{(SB7)}$$

It is clear that combining equations (SB6) and (SB7) gives condition (SB4). This shows that if X and Y are independent, then the ANM is reversible and hence not identifiable.

If the function f is a linear function $ax + b$ then the ANM is reversible. Specifically, assume that $Y = f(X) + N$, where $f(x) = ax + b$ is a bijective and X is uniformly distributed, then ANM is reversible. Now we check that under these conditions, the ANM satisfies equation (SB4).

It is clear that under the ANM, we have

$$P(x, y) = P(x)n(y - f(x)) \quad . \tag{SB8}$$

Assume $g(y) = f^{-1}(y) = \frac{y-b}{a}$. Define the reverse of the ANM:$Y = f(X) + N$ as

$$X = g(Y) + \widetilde{N}, \tag{SB9}$$

which implies

$$X = \frac{Y-b}{a} + \widetilde{N}. \tag{SB10}$$

Let $\widetilde{N} = k$. Multiplying both sides of equation (SB10) by a gives

$aX = Y - b + k$ or

$$Y = aX + b - ak = f(X) + N, \tag{SB11}$$

where

$$N = -ak = b - (ak + b) = b - f(k). \tag{SB12}$$

Therefore, we have

$$P(x, y) = P(Y = y)P(X = x|Y = y) = q(y)P(X = g(y) + k)). \tag{SB13}$$

However,

$$P(X = g(y) + k)) = P(\widetilde{N} = k) = \tilde{n}(k). \quad \text{(SB14)}$$

Using equations (SB11), (SB12) and (SB14), we obtain

$$\tilde{n}(k) = P(Y = f(x) - ak) = P(N = -ak) = P\big(N = b - f(k)\big) = n(b - f(k)). \quad \text{(SB15)}$$

Note that

$$b - f(k) = N = y - f(x). \quad \text{(SB16)}$$

Substituting equation (SB9) and $\widetilde{N} = k$ into equation (SB16), we obtain

$$b - f(k) = y - f(g(y) + k). \quad \text{(SB17)}$$

Substituting equation (SB17) into equation (SB15) yields

$$\tilde{n}(k) = n\big(b - f(k)\big) = n(y - f(g(y) + k)) = n(y - f(x)). \quad \text{(SB18)}$$

Note that

$x = g(y) + k$ or

$k = x - g(y).$,

which implies

$$\tilde{n}(k) = \tilde{n}(x - g(y)) \ . \quad \text{(SB19)}$$

Combining equations (SB9), (SB13), (SB14), (SB18) and (SB19) gives

$$P(x, y) = q(y)\tilde{n}\big(x - g(y)\big) = q(y)n\big(y - f(x)\big) = P(x)n(y - f(x)),$$

which proves the condition (SB4).

Assume that supp X is defined as supp $X \coloneqq \{k|P(X=k)>0\}$. In general, the necessary condition for the reversibility of the ANM is that the product of #supp X and #supp N can be divided by #supp Y (Peters et al. 2011).
