# Supplementary material for: Application of Causal Inference to Genomic Analysis: Advances in Methodology
Source: Front Genet. 2018 Jul 10;9:238. doi: 10.3389/fgene.2018.00238 (PMC6048229; doi:10.3389/fgene.2018.00238)
Supplement: Supplementary file 3 [file Presentation_3.PDF]

Supplementary Note C

Examples Illustrate Causation and Association

Let X be an indicator variable for the genotypes of a SNP, taking values of 0, 1 and 2 to indicate three genotypes, and Y be a binary trait to represent the disease status of the individual where $Y = \begin{cases} 1, & \text{disease} \\ 0, & \text{normal} \end{cases}$. Define the joint probabilities

$$a_0 = P(X = 0, Y = 0), a_1 = P(X = 1, Y = 0), a_2 = P(X = 2, Y = 0), \tag{SC1}$$

$$b_0 = P(X = 0, Y = 1), b_1 = P(X = 1, Y = 1), b_2 = P(X = 2, Y = 1). \tag{SC2}$$

In order for the variables to be nondegenerate, we assume $0 < P(X = 0) = a_0 + b_0 < 1$, $0 < P(X = 1) = a_1 + b_1 < 1$ and $0 < P(X = 2) = a_2 + b_2 < 1$.

Define the penetrance as

$$f_0 = P(Y = 1|X = 0), f_1 = P(Y = 1|X = 1), f_2 = P(Y = 1|X = 2). \tag{SC3}$$

An additive noise model for the disease is defined as

$$Y = f(X) + N, \perp\!\!\!\perp X\ , \tag{SC4}$$

where Y is 2-cyclic, X is 3-cyclic and N is 2-cyclic, f is a general integer function. The noise N includes environmental factors and other genetic factors that are not included in X. The ANM for the disease is similar to the classic decomposition of phenotypic value (Falconer 1989):

$$P = G + E, \tag{SC5}$$

where P is a phenotypic value, G is a genotypic value and E represents environmental contribution.

If the association of the SNP with the trait is used to measure the dependence between the SNP (X) and the trait (Y), then no association indicates that the genotype variable X and the trait variable Y are independent. In terms of penetrance, we have

$$P(Y = 1|X = 0) = P(Y = 1|X = 1) = P(Y = 1|X = 2), \qquad \text{(SC6)}$$

which implies that no association is equal to

$$f_0 = f_1 = f_2 = f^*. \qquad \text{(SC7)}$$

The nonlinear integer function f can be divided into two cases: constant and nonconstant. We first consider the constant case.

Nonlinear integer function f is constant.

If $f(x)$ is constant c, then using the model (SC4) we have $Y = c + N$, which implies

$$P(Y = y|X) = P(c + N = y|X) = P(N = y - c|X). \qquad \text{(SC8)}$$

Since N and X are independent, we have

$$P(N = y - c|X) = P(N = y - c) = P(N + c = y) = P(Y = y). \qquad \text{(SC9)}$$

Combining equations (SC8) and (SC9), we show that X and Y are independent. Therefore, the ANM is reversible. For the convenience of presentation, we assume that the ANM is reduced to

$$Y = N. \qquad \text{(SC10)}$$

The condition $N \perp\!\!\!\perp X$ requires that

$$P(N = 1|X = 0) = P(N = 1|X = 1) = P(N = 1|X = 2). \quad \text{(SC11)}$$

Note that

$$P(N = 1|X = 0) = P(Y = 1|X = 0) = \frac{b_0}{a_0 + b_0}. \quad \text{(SC12)}$$

Similarly, we have

$$P(N = 1|X = 1) = \frac{b_1}{a_1 + b_1} \text{ and } P(N = 1|X = 2) = \frac{b_2}{a_2 + b_2}. \quad \text{(SC13)}$$

Combining equations (SC11), (SC12) and (SC13) gives

$$\frac{a_0}{a_0 + b_0} = \frac{a_1}{a_1 + b_1} = \frac{a_2}{a_2 + b_2} \text{ or}$$

$$a_0 b_1 = a_1 b_0, a_0 b_2 = a_2 b_0, a_1 b_2 = a_2 b_1. \quad \text{(SC14)}$$

Using equation (SC11), we obtain

$$P(N = 1|X = 0) = P(Y = 1|X = 0) = f_0. \quad \text{(SC15)}$$

Similarly, we have

$$P(N = 1|X = 1) = f_1 \text{ and } P(N = 1|X = 2) = f_2. \quad \text{(SC16)}$$

We prove that the ANM from X to Y if and only if $f_0 = f_1 = f_2$.

Similarly, we can show that for an ANM from Y to X:

$$X = c + \tilde{N},\ \tilde{N} \perp\!\!\!\perp Y,$$

We obtain

$$P(\tilde{N}=0|Y=0)=\frac{P(X=0)}{P(Y=0)}(1-f_0),$$

$$P(\tilde{N}=0|Y=1)=\frac{P(X=0)}{P(Y=1)}f_0,$$

$$P(\tilde{N}=1|Y=0)=\frac{P(X=1)}{P(Y=0)}(1-f_1),$$

$$P(\tilde{N}=1|Y=1)=\frac{P(X=1)}{P(Y=1)}f_1,$$

$$P(\tilde{N}=2|Y=0)=\frac{P(X=2)}{P(Y=0)}(1-f_2),$$

$$P(\tilde{N}=2|Y=1)=\frac{P(X=2)}{P(Y=1)}f_2.$$

This shows that the ANM from Y to X if and only if $f_0=f_1=f_2$. Therefore, if f is constant, no causation if and only if no association.

Nonlinear integer function f is nonconstant.

Taking Peters et al.' (2011) approach, without loss of generality, we assume the following ANM from X to Y:

$$Y=X+N,\ N \perp\!\!\!\perp X. \quad \text{(SC17)}$$

The independence condition $N \perp\!\!\!\perp X$ requires

$$P(N=1|X=0)=P(N=1|X=1)=P(N=1|X=2). \quad \text{(SC18)}$$

Note that using $Y=X+N$, we obtain

$$P(N=1|X=0)=P(Y=1|X=0)=f_0, \quad \text{(SC19)}$$

$$P(N = 1|X = 1) = P(Y = 0|X = 1) = 1 - f_1, \quad \text{(SC20)}$$

$$P(N = 1|X = 2) = P(Y = 1|X = 2) = f_2. \quad \text{(SC21)}$$

Using equations (SC1) and (SC2), we can also show that

$$P(N = 1|X = 0) = \frac{b_0}{a_0 + b_0}, \quad \text{(SC22)}$$

$$P(N = 1|X = 1) = \frac{a_1}{a_1 + b_1}, \quad \text{(SC23)}$$

$$P(N = 1|X = 2) = \frac{b_2}{a_2 + b_2}. \quad \text{(SC24)}$$

Substituting equations (SC19)-(SC24) into equation (SC18) gives the condition for the ANM from X to Y:

$$f_0 = 1 - f_1 = f_2, \quad \text{(SC25)}$$

or

$$\frac{b_0}{a_0 + b_0} = \frac{a_1}{a_1 + b_1} = \frac{b_2}{a_2 + b_2}, \quad \text{(SC26)}$$

or

$$a_0 a_1 = b_0 b_1, a_2 b_0 = a_0 b_2, a_1 a_2 = b_1 b_2 \ . \quad \text{(SC27)}$$

Now consider the ANM from Y to X. Without loss of generality, we assume the ANM:

$$X = Y + \tilde{N}\,,\ Y \perp\!\!\!\perp \tilde{N}. \quad \text{(SC28)}$$

The condition $Y \perp\!\!\!\perp \tilde{N}$ requires

$$P\big(\tilde{N} = 1 \big| Y = 0\big) = P(\tilde{N} = 1|Y = 1). \quad \text{(SC29)}$$

Using equation $X = Y + \tilde{N}$, we obtain

$$P\left(\tilde{N} = 1 \middle| Y = 0\right) = P(X = 1|Y = 0) = \frac{P(X=1)}{P(Y=0)}(1 - f_1), \qquad \text{(SC30)}$$

$$P\left(\tilde{N} = 1 \middle| Y = 1\right) = P(X = 2|Y = 1) = \frac{P(X=2)}{P(Y=1)} f_2, \qquad \text{(SC31)}$$

or

$$P\left(\tilde{N} = 1 \middle| Y = 0\right) = \frac{a_1}{a_0+a_1+a_2},$$

$$P\left(\tilde{N} = 1 \middle| Y = 1\right) = \frac{b_2}{b_0+b_1+b_2}. \qquad \text{(SC32)}$$

Therefore,

$$\frac{P(X=1)}{P(Y=0)}(1 - f_1) = \frac{P(X=2)}{P(Y=1)} f_2 \text{ or} \qquad \text{(SC33)}$$

$$a_1 b_0 + a_1 b_1 = a_0 b_2 + a_2 b_2. \qquad \text{(SC34)}$$

Similarly, we obtain

$$P\left(\tilde{N} = 0 \middle| Y = 0\right) = \frac{P(X=0)}{P(Y=0)}(1 - f_0) = \frac{a_0}{a_0+a_1+a_2},$$

$$P\left(\tilde{N} = 0 \middle| Y = 1\right) = \frac{P(X=1)}{P(Y=1)} f_1 = \frac{b_1}{b_0+b_1+b_2},$$

$$P\left(\tilde{N} = 2 \middle| Y = 0\right) = \frac{P(X=2)}{P(Y=0)}(1 - f_2) = \frac{a_2}{a_0+a_1+a_2},$$

$$P\left(\tilde{N} = 2 \middle| Y = 1\right) = \frac{P(X=1)}{P(Y=1)} f_1 = \frac{b_1}{b_0+b_1+b_2},$$

$$a_0 b_0 + a_0 b_2 = a_1 b_1 + a_2 b_1,\ a_2 b_0 + a_2 b_2 = a_0 b_1 + a_1 b_1.$$

In summary, $f_0 = 1 - f_1 = f_2$ or $a_0 a_1 = b_0 b_1, a_2 b_0 = a_0 b_2, a_1 a_2 = b_1 b_2$ leads to an ANM from X to Y and $\frac{P(Y=1)}{P(Y=0)} = \frac{P(X=1)}{P(X=0)} \frac{f_1}{1-f_0}, \frac{P(Y=1)}{P(Y=0)} = \frac{P(X=1)}{P(X=2)} \frac{f_1}{1-f_2}, \frac{P(Y=1)}{P(Y=0)} = \frac{P(X=2)}{P(X=1)} \frac{f_2}{1-f_1}$, or $a_1 b_0 + a_1 b_1 = a_0 b_2 + a_2 b_2$, $a_0 b_0 + a_0 b_2 = a_1 b_1 + a_2 b_1$, $a_2 b_0 + a_2 b_2 = a_0 b_1 + a_1 b_1$leads to an ANM from Y to X.

Example 2 (Figure S2)

Consider two variants in a short genomic region. One variant is a risk variant and another variant is a protection variant. Assume that risk allele or protection allele is a rare allele. The variants are coded as follows:

$$X = \begin{cases} 1, & \text{at least one rare allele at two loci} \\ 0, & \text{otherwise} \end{cases}.$$

Define an ANM:

$$Y = Y_1 + Y_2, \quad \text{(SC35)}$$

where

$$Y_1 = f_1(X) + N, Y_2 = f_2(X) + \tilde{N}, N \perp\!\!\!\perp X, \tilde{N} \perp\!\!\!\perp X. \quad \text{(SC36)}$$

$N \perp\!\!\!\perp X$ condition requires

$$P(N = 1|X = 0) = P(Y_1 = 1|X = 0) = f_0, \quad \text{(SC38)}$$

$$P(N = 1|X = 1) = P(Y_1 = 0|X = 1) = 1 - f_1, \quad \text{(SC39)}$$

$$f_0 = 1 - f. \quad \text{(SC40)}$$

Similarly, $\tilde{N} \perp\!\!\!\perp X$ condition requires

$$P(\tilde{N} = 1 | X = 0) = P(Y_2 = 1|X = 0) = h_0, \quad \text{(SC41)}$$

$$P(\tilde{N} = 1 | X = 1) = P(Y_2 = 0|X = 1) = 1 - h_1. \quad \text{(SC42)}$$

$$h_0 = 1 - h_1. \quad \text{(SC43)}$$

Now we calculate the total genetic effect (Figure S2). Using total probability formula, we obtain $P(Y = 1|X = 0) = P(Y_1 = 1, Y_2 = 0|X = 0) + P(Y_1 = 0, Y_2 = 1|X = 0)$

$$= P(Y_1 = 1|X = 0)P(Y_2 = 0|X = 0) + P(Y_1 = 0|X = 0)P(Y_2 = 1|X = 0)$$

$$= f_0(1 - h_0) + (1 - f_0)h_0. \quad \text{(SC44)}$$

$$P(Y = 1|X = 1) = P(Y_1 = 1, Y_2 = 0|X = 1) + P(Y_1 = 0, Y_2 = 1|X = 1)$$

$$= P(Y_1 = 1|X = 1)P(Y_2 = 0|X = 1) + P(Y_1 = 0|X = 1)P(Y_2 = 1|X = 1)$$

$$= f_1(1 - h_1) + (1 - f_1)h_1. \quad \text{(SC45)}$$

Using equations (SC39), (SC43), (SC44) and (SC45), we can show that

$$P(Y = 1|X = 0) = P(Y = 1|X = 1) = f_0h_1 + f_1h_0. \quad \text{(SC46)}$$

This shows that there is no association in the genomic region, but has causations in the risk variant locus and protection variant locus.
