# Supplementary figures and images for: Application of Causal Inference to Genomic Analysis: Advances in Methodology
Source: Front Genet. 2018 Jul 10;9:238. doi: 10.3389/fgene.2018.00238 (PMC6048229; doi:10.3389/fgene.2018.00238)

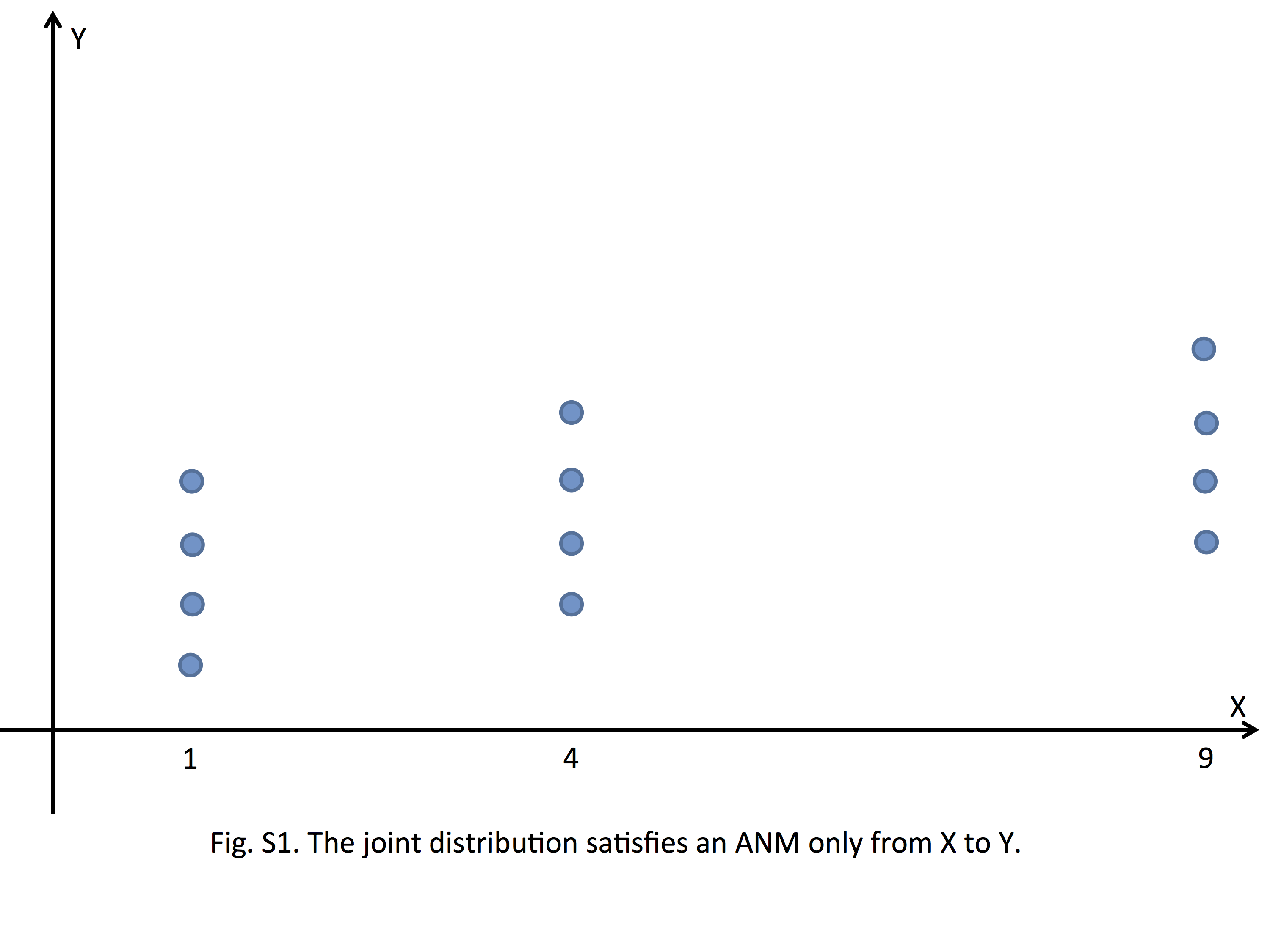

Supplement: Supplementary file 4 [file Image_1.tiff]

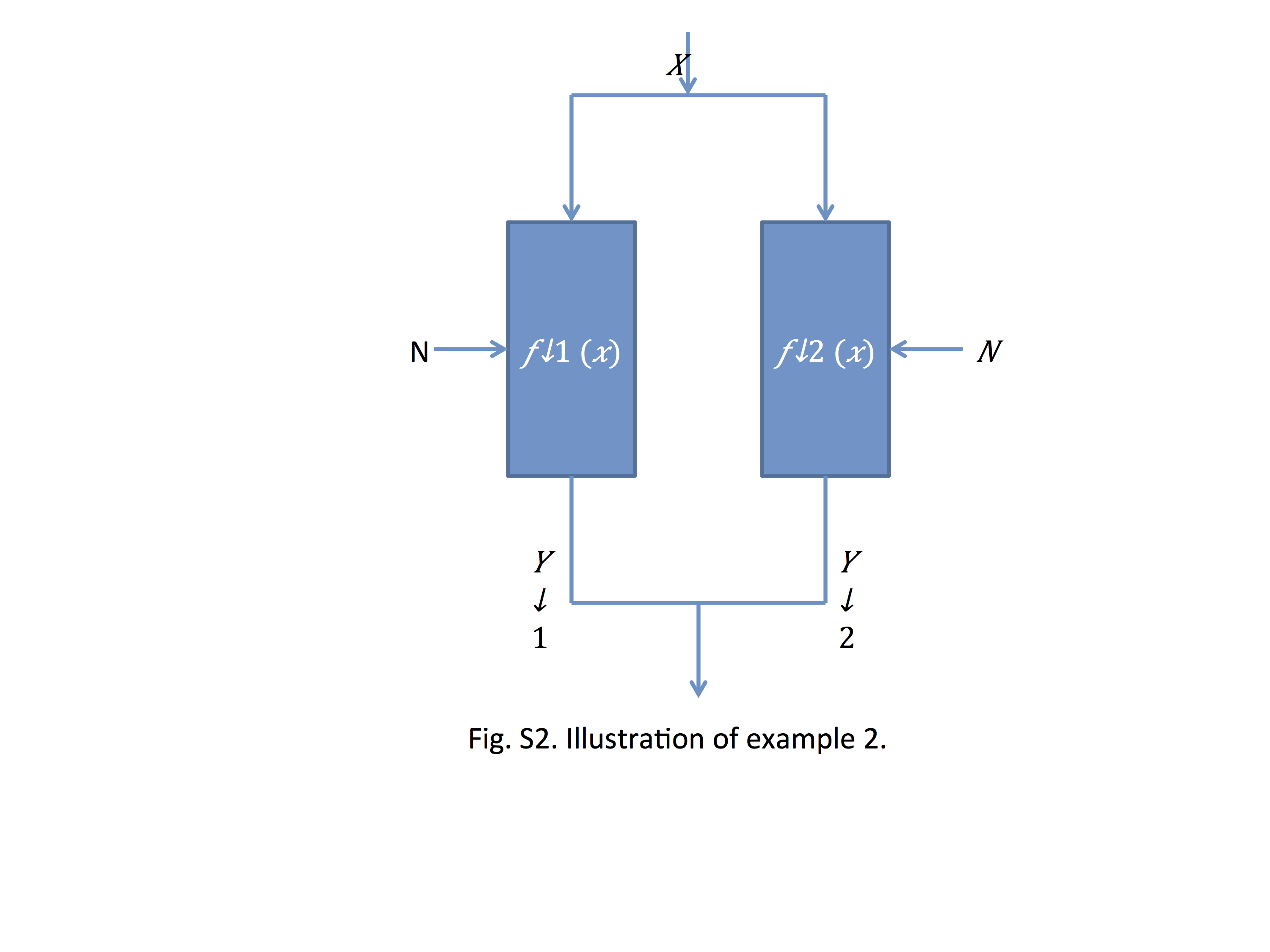

Supplement: Supplementary file 5 [file Image_2.tiff]
